# Generation of *ST6GAL1/ST3GAL4* double-knockout Landrace pigs with reduced respiratory sialylated glycans and decreased influenza A virus susceptibility of derived fibroblasts *in vitro*

**DOI:** 10.7555/JBR.39.20250300

**Published:** 2026-09-28

**Authors:** Lin Li, Shuyi Fang, Xiaoxue Li, Jingjing Liu, Wenhan Lu, Qinyuan Li, Jianghao Chang, Yilin Yuan, Bin Fang, Ying Wang, Yi Rao, Haiyuan Yang, Yifan Dai

**Affiliations:** 1 Department of Medical Genetics, School of Basic Medical Science, Nanjing Medical University, Nanjing, Jiangsu 211166, China; 2 Jiangsu Province Key Laboratory of Biological Therapy for Organ Failure, Nanjing Medical University, Nanjing, Jiangsu 211166, China; 3 Gcreatene (Suzhou) Biotechnology Co., Ltd., Suzhou Industrial Park, Suzhou, Jiangsu 215000, China; 4 Peking-Tsinghua Center for Life Sciences, Peking University, Beijing 100871, China

**Keywords:** gene-edited pigs, influenza A virus, *ST6GAL1*, *ST3GAL4*

## Abstract

Pigs are important hosts in the ecology and cross-species transmission of influenza A viruses because their respiratory tract can contain both human-type α2,6-linked and avian-type α2,3-linked sialylated glycans. These glycan structures may allow pigs to support infection by influenza viruses of different host origins and can facilitate viral reassortment when coinfection occurs. Here, we used CRISPR/Cas9-mediated gene editing to disrupt the porcine *ST6GAL1* and *ST3GAL4* genes, which encode sialyltransferases involved in the biosynthesis of α2,6-linked and α2,3-linked sialylated glycans, respectively. We generated *ST6GAL1/ST3GAL4* double-knockout Landrace pigs and observed markedly reduced *Sambucus nigra* agglutinin (SNA)- and *Maackia amurensis* lectin Ⅱ (MAL Ⅱ)-reactive sialylated glycans in the respiratory tract. Fibroblasts derived from the double-knockout pigs showed reduced susceptibility to H1N1, H3N2, and H9N2 influenza A virus infection *in vitro*. Transcriptomic analysis further revealed distinct gene-expression profiles between knockout and wild-type fibroblasts before and after H1N1 infection. Complementation of *ST6GAL1* and *ST3GAL4* restored sialylated glycan expression and increased viral susceptibility in cultured cells, supporting a causal contribution of these sialyltransferases to influenza A virus infection. Further *in vivo* challenge, transmission, safety, and independent-founder studies are required to determine whether this strategy can reduce influenza A virus infection or transmission in live pigs.

## Introduction

Influenza represents a significant global health threat, responsible for approximately one billion cases of seasonal illness each year, including 3–5 million severe cases and causing 290000 to 650000 respiratory deaths worldwide^[1]^. The propensity of influenza viruses for antigenic drift and viral reassortment enables them to evade previously acquired host immunity and facilitates their continued transmission^[2–3]^. Influenza A virus (IAV), the primary driver of seasonal epidemics and pandemics, poses a continuous threat to public health. The infectivity of IAV begins with the binding of its surface glycoprotein, hemagglutinin (HA), to sialylated glycan attachment determinants on host cells, with HA serving as a critical determinant of viral pathogenicity^[4–5]^. Avian influenza A virus strains typically prefer α2,3-linked sialylated glycans, whereas human strains predominantly utilize α2,6-linked sialylated glycans for attachment^[6]^. These differences in glycan-binding preference contribute to host-range barriers and can restrict the direct transmission of avian IAVs to humans. Because pigs can harbor influenza viruses of avian, swine, and human origin, they may serve as intermediate hosts in IAV ecology and as potential mixing hosts in which reassortment can occur^[7]^. Coinfection of a host cell by influenza viruses of different origins can result in the exchange of viral genome segments and the emergence of novel reassortant viruses^[8–9]^. However, pigs are not the only hosts capable of supporting influenza virus reassortment, and the contribution of any particular host to the emergence and transmission of novel viruses depends on viral, host, ecological, and epidemiological factors.

Current strategies for preventing and treating influenza primarily depend on prophylactic vaccination and antiviral therapy^[10]^. However, the rapid evolution of IAVs often leads to antiviral resistance^[11–14]^. Seasonal influenza vaccines require periodic updating because of antigenic evolution, and their effectiveness varies according to the match between vaccine strains and circulating viruses^[15–17]^. Moreover, the time required to develop new vaccines can significantly delay responses to emerging strains, undermining public health interventions and exacerbating socioeconomic impacts. Influenza also affects animal health, agricultural production, and the economy^[18–19]^. In animal populations, influenza control may require continuous surveillance, vaccination, biosecurity measures, and management of infected or exposed animals^[20]^. These challenges highlight the importance of investigating complementary approaches that target host determinants of influenza susceptibility.

CRISPR/Cas9 is an efficient gene-editing technology that has been widely applied in biotechnology, agriculture, and biomedical research^[21]^. CRISPR/Cas9-mediated editing can generate targeted mutations without intentional integration of an exogenous transgene. In some jurisdictions, animals carrying such edits may be subject to regulatory frameworks different from those applied to conventional transgenic animals. However, regulatory classification and safety-evaluation requirements vary substantially among countries and applications, and the absence of an introduced transgene does not by itself establish reduced biological risk^[22–25]^. Several genetically modified porcine models have been investigated in the context of influenza susceptibility. These include *TMPRSS2*-knockout pigs, in which the host transmembrane serine protease involved in the proteolytic activation of influenza hemagglutinin is disrupted^[26–27]^, and *Mx1*-transgenic pigs expressing a broad-spectrum myxovirus-resistance factor^[28–29]^. These studies demonstrate the potential of host-directed genetic modification for investigating viral susceptibility in livestock. Nevertheless, host factors involved in viral attachment, entry, replication, and innate antiviral defense have distinct biological functions and may produce different effects on animal physiology and viral infection.

Sialyltransferases are key enzymes involved in the biosynthesis of terminal sialylated glycans. *ST6GAL1* encodes β-galactoside α2,6-sialyltransferase 1, which contributes to the biosynthesis of α2,6-linked sialylated glycans, whereas *ST3GAL4* encodes α2,3-sialyltransferase 4, which contributes to the biosynthesis of α2,3-linked sialylated glycans. These sialylated glycan structures can serve as attachment determinants for IAV HA, although they do not constitute the complete viral entry machinery. Previous studies have suggested that disruption of ST6GAL1- or ST3GAL4-dependent sialylation can reduce IAV susceptibility in mammalian cells^[30–32]^.

In the present study, we used CRISPR/Cas9-mediated gene editing and somatic cell nuclear transfer (SCNT) to generate *ST6GAL1/ST3GAL4* double-knockout Landrace pigs. We characterized the edited animals and examined the distribution of *Sambucus nigra* agglutinin (SNA)- and *Maackia amurensis* lectin Ⅱ (MAL Ⅱ)-reactive sialylated glycans in their respiratory tracts. Because live gene-edited pigs were not subjected to influenza virus challenge, the antiviral phenotype was evaluated in fibroblasts derived from the edited pigs. We assessed the susceptibility of these cells to H1N1, H3N2, and H9N2 IAV infection and performed transcriptomic analysis following H1N1 infection. In addition, complementation of *ST6GAL1* and *ST3GAL4* was used to examine whether restoration of sialylation was accompanied by increased viral susceptibility.

## Materials and methods

### Animals, cell lines, and viruses

Landrace pigs were purchased from Zhengda Co., Ltd. (Huai'an, China) and raised in a large animal facility affiliated with Nanjing Medical University. The pigs were fed chow diets purchased from Chia Tai Co., Ltd. (Huai'an, China) twice daily in accordance with standard pig husbandry procedures. Age-matched wild-type Landrace pigs with identical feeding conditions and housing environment were used as control groups for tissue lectin staining; wild-type pigs were all male, with respiratory tract tissues collected at 17 months of age consistent with surviving knockout piglets. All experiments in this study complied with the guidelines of the Institutional Animal Care and Use Committee (IACUC) of Nanjing Medical University under approved protocol numbers IACUC-1901015 and IACUC-1901043.

Wild-type porcine fetal fibroblasts (PFFs) and *ST6GAL1*^−/−^/*ST3GAL4*^−/−^ porcine fibroblasts were isolated in our laboratory. The newborn pig trachea epithelial (NPTr) cell line was kindly provided by Professor Hongbo Zhou from Huazhong Agricultural University. The Madin-Darby canine kidney (MDCK) cell line and the H1N1, H3N2, and H9N2 influenza viruses were gifts from Professor Jihui Ping at Nanjing Agricultural University. The NPTr and MDCK cell lines were cultured in DMEM (Cat. #11965092, Gibco, Grand Island, NY, USA) supplemented with 10% fetal bovine serum (Cat. #A5670801, Gibco) at 37 ℃ under 5% CO_2_ for 24 h.

### Isolation of Landrace PFFs

Landrace PFFs were isolated from the skin of Landrace pig fetuses at 34–38 days of gestation. The harvested tissue was digested with DMEM containing 200 U/mL collagenase (Cat. #1148090, Sigma-Aldrich, St. Louis, MO, USA) at 37 ℃ for 1 h, with periodic pipetting and visual inspection until tissue dissolution was complete. The digestion was terminated by adding DMEM (Cat. #11965092, Gibco) supplemented with 16% fetal bovine serum (Cat. #A5670801, Gibco). After centrifugation, the cell pellet was resuspended in DMEM complete medium and cultured at 38.5 ℃ under 5% CO_2_.

### Construction of *ST6GAL1*^−/−^/*ST3GAL4*^−/−^ PFFs

The *ST6GAL1/ST3GAL4* gene-targeting plasmid was constructed by integrating four single-guide RNAs (sgRNAs) (two targeting *ST6GAL1* and two targeting *ST3GAL4*; ***Fig. 1A***) into the pX330 plasmid backbone. It was then co-transfected with the pHY58 puromycin selection plasmid at a 5∶1 ratio into 5 × 10^5^ Landrace PFFs using the Basic Fibroblast Nucleofection Kit (Cat. #VPI-1002, Lonza, Cologne, Germany) and nucleofection program U-023. Following electroporation, the cells were plated at a density of 120 cells per 4× microscopic field in 10 cm dishes and cultured in DMEM complete medium containing 1× penicillin/streptomycin (Cat. #0378016, Gibco) at 38.5 ℃ under 5% CO_2_ for 24 h. Once the cells had adhered, selection was initiated using medium containing 1 μg/mL puromycin (Cat. #A1113803, Invitrogen, Carlsbad, CA, USA). Through medium replacement every 48 h with progressively reduced puromycin concentrations, resistant clones were isolated at a maintenance concentration of 0.2 μg/mL puromycin and seeded into 24-well plates, and subsequently passaged to 6-well plates. One-fourth of the cells were reserved for PCR screening (PCR amplification primers are listed in ***Supplementary Table 1***), while the remaining cells were cryopreserved for subsequent SCNT. The PCR amplification products were subcloned into the pMD18-T vector (Cat. #6011, Takara Bio, Tokyo, Japan), and 10–15 individual clones were randomly selected for sequencing verification.

**Figure 1:**
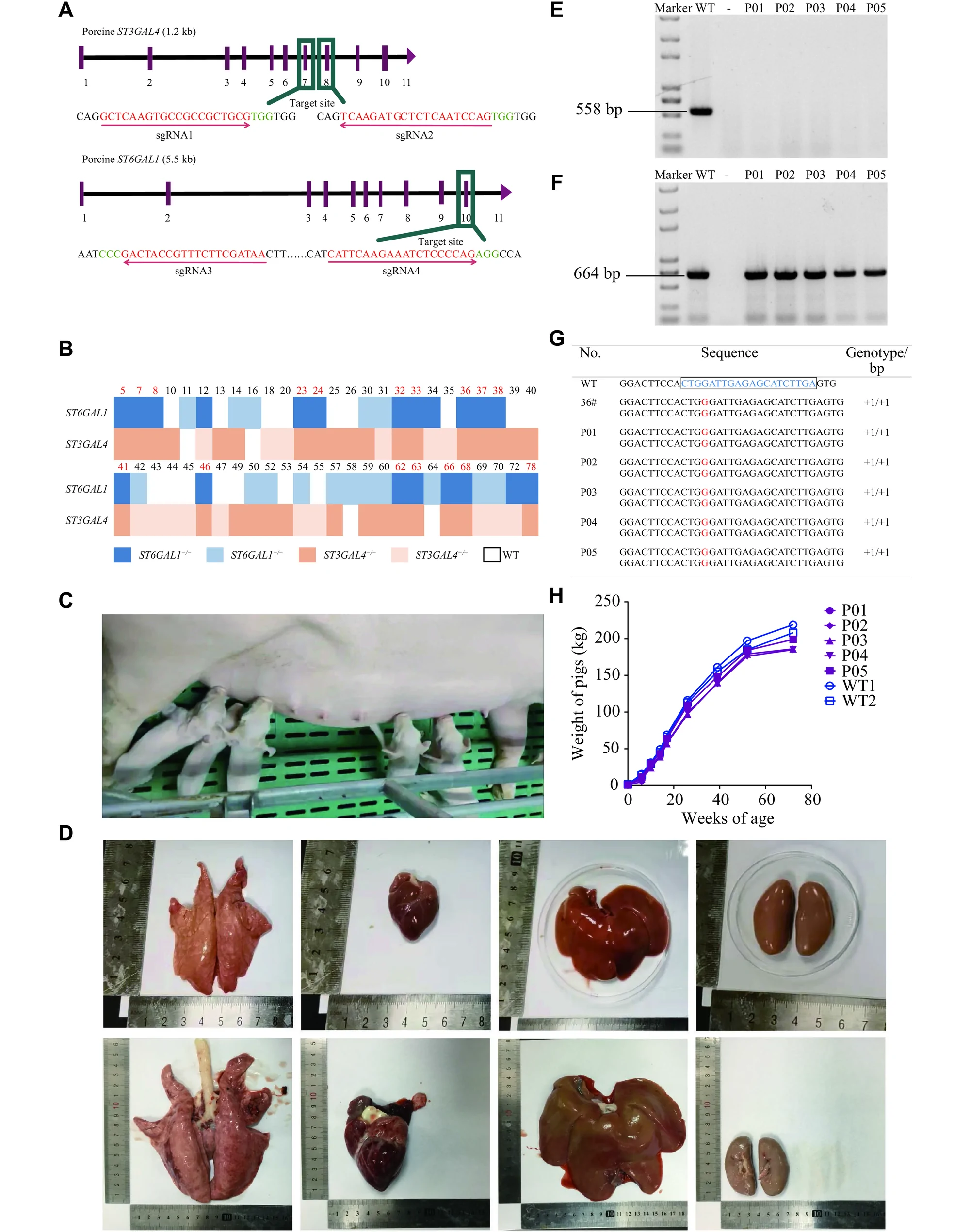
Construction of *ST6GAL1*^−/−^/*ST3GAL4*^−/−^ Landrace pigs. A: Schematic diagram of single-guide RNAs (sgRNAs) targeting the *ST3GAL4* and *ST6GAL1* gene loci. The targeting region is depicted with sgRNAs marked in red and protospacer adjacent motif (PAM) marked in green. B: Summary of genotyping results for *ST3GAL4* and *ST6GAL1* gene-targeted cell colonies. C: Photograph of *ST6GAL1*^−/−^/*ST3GAL4*^−/−^ Landrace pigs. D: Anatomical diagram of piglets P01/P02. The top row shows, in sequence, the lung, heart, liver, and kidney of piglet P01 (aged 5 days at dissection). The bottom row shows, in sequence, the lung, heart, liver, and kidney of piglet P02 (aged 41 days at dissection). E: PCR amplification of the *ST6GAL1* gene in cloned piglets. No positive amplification bands were detected in the *ST6GAL1* targeting fragments of cloned piglets. F: PCR amplification of the *ST3GAL4* gene in cloned piglets. G: Genotypes of cloned piglets. The *ST3GAL4* sequence from wild-type (WT) Landrace pigs is shown as a reference at the top. The sgRNA target site is highlighted in blue, and the insertion of a single guanine (G) base in the cloned piglets is indicated in red. H: Growth curve of body weight in gene-knockout (P01–P05) and age-matched wild-type (WT1, WT2) pigs.

### SCNT and embryo transfer

The selected cumulus-oocyte complexes with intact morphology were collected from slaughterhouse-derived porcine ovaries and cultured in *in vitro* maturation medium at 38.5 ℃ under 5% CO_2_ for 42 h. Upon maturation, the oocytes were subjected to enzymatic denudation and precise enucleation using polar body-guided micromanipulation. Subsequently, genetically modified *ST6GAL1*^−/−^/*ST3GAL4*^*−/−*^donor cells were introduced into the enucleated oocytes. The reconstructed complexes were electrofused using optimized parameters (140 V/mm, 30 μs) and cultured in embryo development medium under mineral oil at 38.5 ℃ for 5 days until they reached the blastocyst stage. For embryo transfer, approximately 150 blastocysts were surgically implanted into the uterus of synchronized recipient sows (on day 5 of estrus), with pregnancy outcomes monitored by ultrasound until parturition.

### Phenotypic characterization of *ST6GAL1*^−/−^/*ST3GAL4*^−/−^ Landrace pigs

#### Genotype identification

Genomic DNA was extracted from ear tissue samples collected from cloned piglets at 30 days postpartum using the ABclonal Rapid Genomic DNA Extraction Kit (Cat. #RK30110, ABclonal, Wuhan, China). PCR amplification was performed with the primers listed in ***Supplementary Table 1***, followed by sequencing of the PCR products to confirm the genotypes of the *ST6GAL1*^−/−^*/ST3GAL4*^−/−^ Landrace pigs.

#### Quantitative reverse transcription-PCR (RT-qPCR) analysis of gene expression

Total RNA was extracted using TRIzol (Cat. #15596026CN, Invitrogen), followed by cDNA synthesis with reverse transcriptase. Gene-specific primers (sequences provided in ***Supplementary Table 2***) were designed to amplify conserved regions of *ST6GAL1* and *ST3GAL4* transcripts, using *GAPDH* as an endogenous reference gene.

#### Immunofluorescence (IF) staining analysis

Cells at 80% confluence were fixed with 4% paraformaldehyde for 30 min, followed by phosphate-buffered saline (PBS) washes. For respiratory tract tissues, including the larynx, trachea, bronchus, and lung, paraffin-embedded tissue sections underwent standard dewaxing, rehydration, and antigen retrieval procedures before IF staining. Sequential blocking was performed using ReadyProbe Streptavidin Blocking Solution (Cat. #R37628, Invitrogen) and ReadyProbes Biotin Blocking Solution (Cat. #R37627, Invitrogen), with intervening PBS washes. Cells and tissues were then incubated with biotinylated lectins MAL Ⅱ (1∶200, Cat. #B-1265-1, Vector Laboratories, Burlingame, CA, USA) or SNA (1∶200, Cat. #B-1305-2, Vector Laboratories) for 1 h, followed by three washes with PBS containing 0.05% Tween-20 (PBST). Alexa Fluor 594-conjugated streptavidin (1∶2000, Cat. #35107ES60, Yeasen, Shanghai, China) was applied in the dark for 1 h, after which cells and tissues were washed with PBST and counterstained with DAPI for nuclear visualization. Imaging was performed using fluorescence microscopy with appropriate excitation/emission filters (594/617 nm for lectins, 358/461 nm for DAPI).

### Viral challenge assay

Cells were seeded into 12-well plates and cultured until they reached 80% confluence. The culture medium was then discarded, and the cells were washed twice with serum-free DMEM. In the meantime, influenza virus stocks were thawed on ice, mixed thoroughly by vortexing, and diluted with pre-chilled PBS to achieve a multiplicity of infection (MOI) of 0.5. After removal of the medium, the diluted viral suspension was added to the cells and incubated at 37 ℃ for 1 h. Subsequently, the viral inoculum was discarded, and the cells were washed with PBS before being covered with a viral maintenance medium. The infected cells were maintained at 37 ℃ with 5% CO_2_ for 96 h. Every 48 h, the supernatant was collected and stored at −80 ℃ for later use in plaque assay quantification. The remaining cells were processed for either indirect immunofluorescence analysis or RNA sequencing (RNA-seq) analysis. For IF staining, cells were fixed and permeabilized before incubation with a primary antibody against influenza A virus nucleoprotein (NP) (1∶1000, Cat. #GTX125989, GeneTex, Irvine, CA, USA) followed by a secondary antibody, Goat Anti-Rabbit IgG H&L (1∶1000, Cat. #ab150077, Abcam, Cambridge, UK).

### Plaque assay

MDCK cells were seeded in 24-well plates and cultured until they reached 100% confluence with tight junctions. The viral supernatant was thawed on ice, vortexed, and then serially diluted 10-fold in cold PBS. A volume of 100 μL of the diluted virus was added to each well (after the cells had been washed with PBS), and the plate was incubated at 37 ℃ with gentle shaking every 15 min. Concurrently, an agarose overlay medium was prepared by mixing melted low-melting-point agarose with 2× DMEM maintenance medium. Following infection, the cells were washed with PBS and overlaid with the prepared agarose medium. The overlay was permitted to solidify at room temperature for 15 min. Finally, the plates were inverted and incubated at 37 ℃ with 5% CO_2_ for 48 h to facilitate plaque formation.

### Construction of *ST6GAL1*/*ST3GAL4* gene complementation PFFs

Primers (***Supplementary Table 1***) with homology arm sequences for the vector plasmid pCAGGS were designed to amplify the cDNA sequences of the *ST6GAL1* and *ST3GAL4* genes, respectively. The amplification products and the pCAGGS plasmid, digested with BsmBI-V2, were purified using a gel extraction kit (Cat. #740611, Takara). Subsequently, a homologous recombination reaction was conducted employing the ClonExpress Ⅱ One Step Cloning Kit (Cat. #C112, Vazyme Biotech, Nanjing, China). The recombinant plasmids pCAGGS-*ST6* and pCAGGS-*ST3* were then transformed into *DH5α* competent cells. Positive clones were screened *via* colony PCR and subsequently verified for the correct insertion and integrity of the target genes through Sanger sequencing.

The pCAGGS-*ST6* and pCAGGS-*ST3* vectors were co-transfected into 5 × 10^5^ *ST6GAL1/ST3GAL4* double-knockout (KO) PFFs using the Basic Fibroblast Nucleofection Kit and nucleofection program U-023. The transfected cells were then cultured in DMEM complete medium at 38.5 ℃ under 5% CO_2_ to allow for gene expression and to generate complemented cells. These complemented cells were subsequently subjected to IF staining and viral challenge assays.

### RNA-seq and bioinformatic analysis

Four experimental groups were included in the RNA-seq analysis: WT sham, WT H1N1-infected, KO sham, and KO H1N1-infected. Each group contained three independently prepared cell-level replicates. The cells were separately cultured and passaged from fibroblasts isolated from one individual pig of the corresponding genotype, and RNA extraction and sequencing-library construction were performed independently for each replicate.

Transcriptome sequencing was performed by OE Biotech Co., Ltd. (Shanghai, China). Libraries were sequenced on an Illumina NovaSeq 6000 platform to generate 150-bp paired-end reads. Raw reads in FASTQ format were processed using fastp v0.20.1 to remove adapters and low-quality reads, yielding clean reads for downstream analysis. Clean reads were mapped to the porcine reference genome using HISAT2 v2.1.0. Gene-level FPKM values were calculated, and read counts per gene were obtained using HTSeq-count v0.11.2. Principal component analysis was performed using R v3.5.1 to evaluate reproducibility among samples. Differential-expression analysis was performed using DESeq2 v1.22.2. Genes with a *Q* value < 0.05 and a fold change > 2 or < 0.5 were considered significantly differentially expressed. Hierarchical clustering of differentially expressed genes (DEGs) was performed using R v3.5.1 to visualize expression patterns across groups and samples.

Gene Ontology, Kyoto Encyclopedia of Genes and Genomes (KEGG) pathway enrichment, Reactome, and WikiPathways enrichment analyses were performed using clusterProfiler v3.10.0 based on the hypergeometric distribution. R v3.5.1 was used to generate column diagrams, chord diagrams, and bubble diagrams for the enriched terms. Gene set enrichment analysis was performed using Gene Set Enrichment Analysis software. Predefined gene sets were ranked according to the magnitude of differential expression between groups, and enrichment at either end of the ranked list was assessed.

### Statistical analysis and replicate definitions

In the current study, all data are presented as mean ± standard deviation. Statistical analyses were performed using GraphPad Prism 9.0.0 software. Comparisons between two groups were performed using an unpaired two-sided Student's *t*-test. Comparisons among three or more groups were performed using one-way analysis of variance followed by Tukey's multiple-comparison test. For viral replication kinetics, group comparisons were performed at each prespecified time point using one-way analysis of variance followed by Tukey's multiple-comparison test. A *P* value < 0.05 was considered statistically significant. Throughout, *n* = 3 denotes independent experimental replicates: for lectin-staining and tissue/cell RNA assays, these were three distinct pigs per genotype; for viral infection, plaque, and immunofluorescence-quantification assays, these were three separate cell-culture and infection procedures performed on different days using the same cell strains. These are considered independent experimental replicates rather than independent animal-level biological replicates.

## Results

### Construction of *ST6GAL1*^−/−^/*ST3GAL4*^−/−^ Landrace pigs using CRISPR/Cas9 technology

The porcine *ST3GAL4* gene (GenBank accession: NC_010451.4) and the *ST6GAL1* gene (NC_010455.5) each contain 11 exons, encoding proteins of 388 and 408 amino acids, respectively. Two sgRNAs were specifically designed to target exon 10 of *ST6GAL1*, while another pair of sgRNAs was designed to target exons 7 and 8 of *ST3GAL4*, using the CRISPOR online tool http://crispor.tefor.net/ (***Fig. 1A***). To optimize co-transfection efficiency and enhance the dual-gene knockout rate, we constructed a multiplex CRISPR system by incorporating all four sgRNAs into a single pX330 plasmid backbone (***Supplementary Fig. 1***). This plasmid, along with the pHY58 puromycin resistance plasmid, was introduced into Landrace PFFs. We harvested 78 puromycin-resistant cell colonies and subjected them to genotyping analysis. The results indicated differential knockout efficiencies: a 46.15% targeting rate (36/78 clones) with a 25.64% biallelic knockout rate (20/78) for *ST6GAL1*, and a more robust 61.54% targeting rate (48/78 clones) with a 41.03% biallelic knockout rate (32/78) for *ST3GAL4*. In total, 16 double-knockout clones (*ST6GAL1*^−/−^/*ST3GAL4*^−/−^) were successfully obtained, demonstrating a dual biallelic knockout efficiency of 20.51% (***Supplementary Tables 3*** and ***4*** and ***Fig. 1B***). These results collectively demonstrate the effectiveness of our multiplex gene editing strategy in generating dual-gene knockout cell lines.

Subsequently, double-knockout Landrace pigs were established through SCNT and embryo transfer into four surrogate sows using pooled *ST6GAL1*^−/−^/*ST3GAL4*^−/−^ colonies (23# and 46# mixed, 33# and 36# mixed) as donor cells. This procedure successfully yielded five live-born male piglets (***Fig. 1C***). Postnatal monitoring revealed that piglets P01 and P02 died spontaneously on the 5^th^ and 41^st^ day, respectively. Immediate necropsy indicated no gross anatomical abnormalities (***Fig. 1D***). The remaining three piglets were raised under standard nursing conditions and reached 17 months of age by the end of the study. No overt abnormalities were observed among the surviving KO pigs during the observation period, despite the KO group exhibiting slightly lower body weights at similar ages (***Fig. 1H***). Sequencing analysis confirmed that all five piglets born *via* SCNT shared the same genotype as the donor cell clone 36#. This genotype was characterized by the complete absence of the *ST6GAL1* target region (***Fig. 1E***) and a single guanine (G) base insertion in the *ST3GAL4* target region (***Fig. 1F*** and ***1G***), which was identical to the genotype of the 36# donor cells (***Supplementary Table 4*** and ***Fig. 1G***). Thus, the animals were derived from a single edited donor-cell genotype, and independent replication using separately generated edited founder lines was not assessed in this study.

### Altered expression of α2,6-linked and α2,3-linked sialylated glycans in *ST6GAL1*^−/−^/*ST3GAL4*^−/−^ Landrace pigs

Previous studies have established that knockout of the *ST6GAL1* and *ST3GAL4* genes in human cells results in a significant reduction in the expression of corresponding sialylated glycans^[30–32]^. Consistent with these findings, quantitative PCR analysis in our study confirmed a substantial downregulation of mRNA expression levels for both *ST6GAL1* and *ST3GAL4* in the gene-edited porcine fibroblasts (***Fig. 2A***). To further validate the changes in sialylated glycan expression, we used biotinylated lectins SNA for α2,6-linked sialylated glycans and MAL Ⅱ for α2,3-linked counterparts in IF staining^[33–34]^. The results indicated a dramatic decrease in the expression of both sialylated glycans on the surface of KO pig cells (***Fig. 2B***).

**Figure 2:**
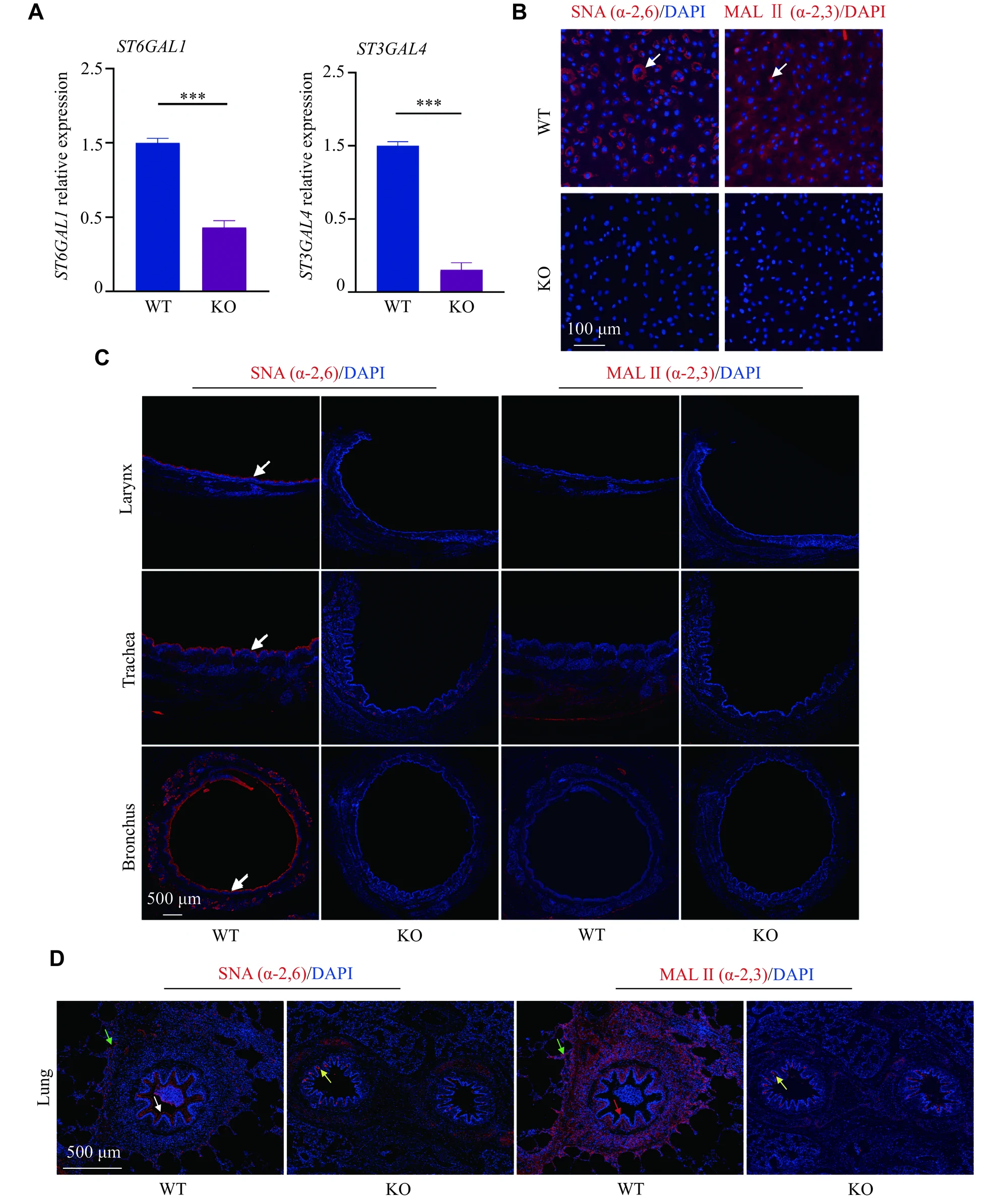
Immunofluorescent detection of α2,6-linked and α2,3-linked sialylated glycans in wild-type and *ST6GAL1^−/−^/ST3GAL4^−/−^* pig fibroblasts/respiratory tissues. A: Quantitative reverse transcription-PCR analysis of *ST6GAL1* and *ST3GAL4* gene expression levels in *ST6GAL1*^−/−^/*ST3GAL4*^−/−^ knockout (KO) and wild-type (WT) pig fibroblasts. Data are presented as mean ± standard deviation (SD); *n* = 3 independent biological replicates, representing fibroblasts isolated from three separate pigs for each genotype group. Statistical significance between WT and KO groups was assessed by unpaired two-sided Student's *t*-test, with ^***^P < 0.001. B: Immunofluorescent detection of α2,6-linked and α2,3-linked sialylated glycans in WT and KO pig fibroblasts. C and D: Immunofluorescent detection of α2,6-linked and α2,3-linked sialylated glycans in WT and KO pig respiratory tract. Panels show the upper respiratory tract including larynx, trachea, bronchus (C), and lung tissue (D). Red indicates SNA (α2,6) or MAL Ⅱ (α2,3) staining, and blue indicates nuclei. White arrows indicate sialylated glycan-positive regions in WT samples (B and C). In panel D, WT pigs exhibited a strong α2,6-linked sialylated glycan signal (white arrow) in bronchiolar epithelium, lacking an α2,3-linked sialylated glycan signal (red arrow); both sialylated glycans were present in WT alveoli (green arrows), but absent in KO pig alveoli; weak signals of both sialylated glycans were detected in the bronchiolar epithelium of KO pigs (yellow arrows). Scale bars, 100 μm (B) and 500 μm (C and D).

To assess the effect of gene knockout on the distribution of α2,6-linked and α2,3-linked sialylated glycans within the respiratory system, immunohistochemical analysis was performed. In wild-type pigs, strong SNA binding signals were detected exclusively in the epithelial layers of the larynx, trachea, and bronchi, with no MAL Ⅱ signal observed (***Fig. 2C***), indicating that α2,6-linked sialylated glycans dominate in these regions. Conversely, in knockout pigs, neither SNA nor MAL Ⅱ binding was detectable in the upper respiratory tract (***Fig. 2C***). In the lower respiratory tract, while wild-type pigs exhibited abundant SNA signals in bronchiolar epithelial cells and minimal detection in the alveolar epithelium, the *ST6GAL1*^−/−^*/ST3GAL4*^−/−^ pigs retained only faint SNA signals in the bronchiolar epithelium. Notably, MAL Ⅱ staining, which was prominent in alveolar and bronchiolar endothelial tissues of wild-type pigs, showed a presence in the bronchiolar epithelial cells of knockout pigs, albeit weakly (***Fig. 2D***). This pattern is consistent with prior reports that α2,3-linked sialylated glycans accumulate and dominate in porcine lower respiratory compartments of wild-type pigs^[33–34]^. In the knockout animals, SNA- and MAL Ⅱ-reactive signals were markedly reduced, although weak residual staining remained in selected bronchiolar and endothelial regions. The altered lectin-binding patterns confirm the functional consequences of *ST6GAL1* and *ST3GAL4* knockout on sialylated glycan distribution throughout the respiratory system.

### Influenza virus challenge assay in *ST6GAL1*^−/−^/*ST3GAL4*^−/−^ pig fibroblasts

We selected three IAV strains—the zoonotic swine H1N1 and H3N2 viruses and the low-pathogenic H9N2 virus—to infect fibroblasts from WT and KO pigs. NPTr cells were used as a positive control. Comparative infection analysis revealed significant differences in cellular responses among the experimental groups. The KO group maintained normal morphology without significant cytopathic effects at both 48 and 72 h post-infection (HPI), whereas WT cells began to display characteristic cytopathic effect features, such as cell clustering, swelling, nuclear pyknosis, and eventual cell death starting at 48 HPI. The NPTr positive controls showed the most severe cytopathic effect, with rapid cell detachment occurring shortly after infection (***Fig. 3A*** and ***Supplementary Fig. 2***).

**Figure 3:**
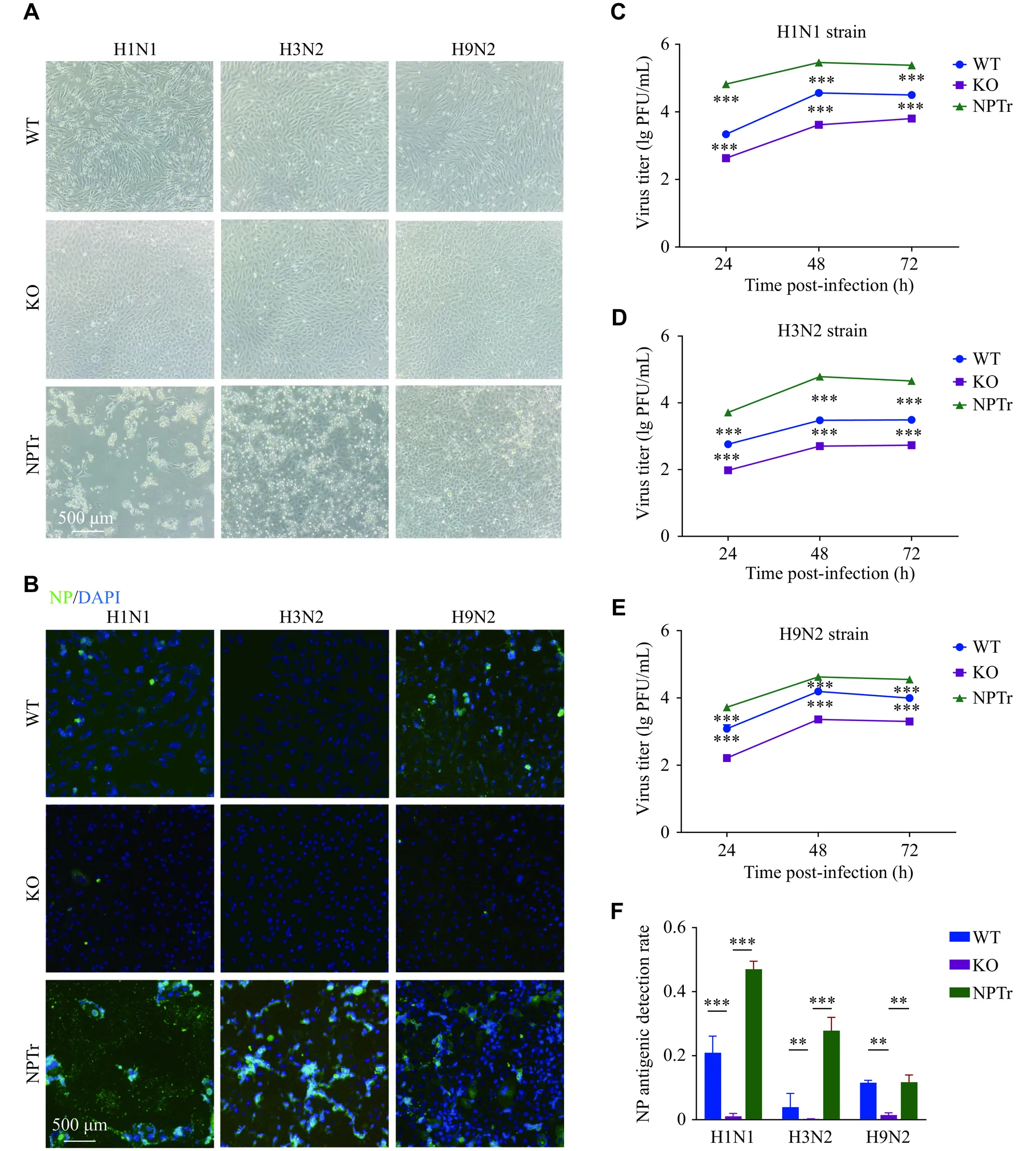
Resistance of *ST6GAL1*^−/−^/*ST3GAL4*^−/−^ Landrace pig cells to influenza A virus (IAV) infection. A: Cytopathic effects induced by H1N1, H3N2, and H9N2 virus infection (multiplicity of infection [MOI] = 0.5) at 48 h post-infection (HPI). Scale bar, 500 μm. B: Immunofluorescent detection of IAV nucleoprotein (NP) in knockout (KO) and wild-type (WT) pig fibroblasts at 48 HPI. Scale bar, 500 μm. NPTr were used as positive controls. Green indicates NP-Alexa Fluor 488, and blue indicates nuclei. C–E: Viral replication kinetics of H1N1, H3N2, and H9N2 influenza viruses in different cell groups as determined by plaque assay (MOI = 0.5). One-way ANOVA followed by Tukey's multiple-comparisons post hoc test was performed separately at each time-point, with the KO group set as the reference for comparisons against the WT and NPTr groups. F: Semiquantitative analysis of influenza A virus NP-specific immunofluorescence intensity at 48 HPI. One-way ANOVA followed by Tukey's multiple-comparisons post-hoc test was used for group comparisons. Data are presented as mean ± standard deviation; *n* = 3 independent cell culture experiments conducted with identical cell batches. ^**^P < 0.01 and ^***^P < 0.001 versus the KO group.

IF staining of influenza A virus NP further validated these observations: qualitative assessment at 24, 48, and 72 HPI indicated that the *ST6GAL1*^−/−^/*ST3GAL4*^−/−^ fibroblasts consistently exhibited lower NP antibody binding across all time points (***Fig. 3B*** and ***Supplementary Fig. 3***). Quantitative analysis of NP-specific fluorescence intensity at 48 HPI confirmed this reduction (***Fig. 3F***). In contrast, the WT fibroblasts and NPTr cells exhibited significantly higher binding at all time points, with NPTr cells displaying particularly strong signals and massive cell detachment.

This partial resistance pattern was consistently observed across all tested influenza subtypes, with H1N1 demonstrating the most pronounced differential infectivity between groups. The viral replication kinetics also supported the above observations (***Fig. 3C–3E***).

### Transcriptome analyses of *ST6GAL1*^−/−^/*ST3GAL4*^−/−^ and WT pig fibroblasts after influenza virus H1N1 infection

To investigate the effect of *ST6GAL1* and *ST3GAL4* gene knockouts on the resistance of Landrace pig fibroblasts to influenza virus, four experimental groups (WT sham, WT H1N1-infected, KO sham, KO H1N1-infected) were included in RNA-seq profiling. Each group contained 3 independent cell-level biological replicates of fibroblasts, which were separately cultured and passaged from fibroblasts isolated from a single individual pig of the corresponding genotype, with RNA extracted and sequencing libraries constructed independently for every replicate. Principal component analysis indicated that the four datasets exhibited high reproducibility, with sample points showing a clear and well-defined clustering trend in the principal component space, demonstrating strong reliability and consistency (***Supplementary Fig. 4A***). Due to the knockouts of the *ST6GAL1* and *ST3GAL4* genes, the KO group exhibited a total of 1280 DEGs compared with the WT group, including 495 upregulated genes and 785 downregulated genes (***Supplementary Fig. 4B***). Upon viral infection, the WT group showed 2914 DEGs (1982 upregulated and 932 downregulated), whereas the KO group only had 2003 DEGs (1521 upregulated and 482 downregulated) (***Supplementary Fig. 4B***). The number of genes meeting the predefined differential-expression thresholds was 31.3% lower in the knockout infected group than in the wild-type infected group.

KEGG pathway analysis indicated that the transcriptional alterations were predominantly enriched in pathways associated with pathogen infection and immune responses (***Fig. 4A*** and ***4B***). Moreover, DEGs in KO pig cells were notably enriched in pathways associated with cardiomyopathy (encompassing dilated and hypertrophic cardiomyopathy) and various cancer pathways, in both sham and infected groups. These cells also exhibited consistent changes in critical signaling transduction pathways, such as extracellular matrix (ECM)-receptor interaction, PI3K-Akt, and Rap1 signaling pathways (***Fig. 4C*** and ***4D***). This persistent alteration implies that the dual knockout of *ST6GAL1* and *ST3GAL4* may reconfigure cellular response capabilities to external stimuli *via* these conserved regulatory networks. Furthermore, H1N1 infection led to significant enrichment of DEGs in the complement and coagulation cascades pathway in KO pig cells compared with WT cells (***Fig. 4D***), highlighting a unique transcriptional response that could contribute to the observed differences in susceptibility to influenza virus infection. Additionally, analysis of the gene expression levels of *ST3GAL* and *ST6GAL* family members showed that the expression of *ST3GAL1* and *ST3GAL5* genes was significantly upregulated in KO pig cells, potentially compensating for the downregulation of *ST3GAL4* gene expression (***Supplementary Fig. 4C***).

**Figure 4:**
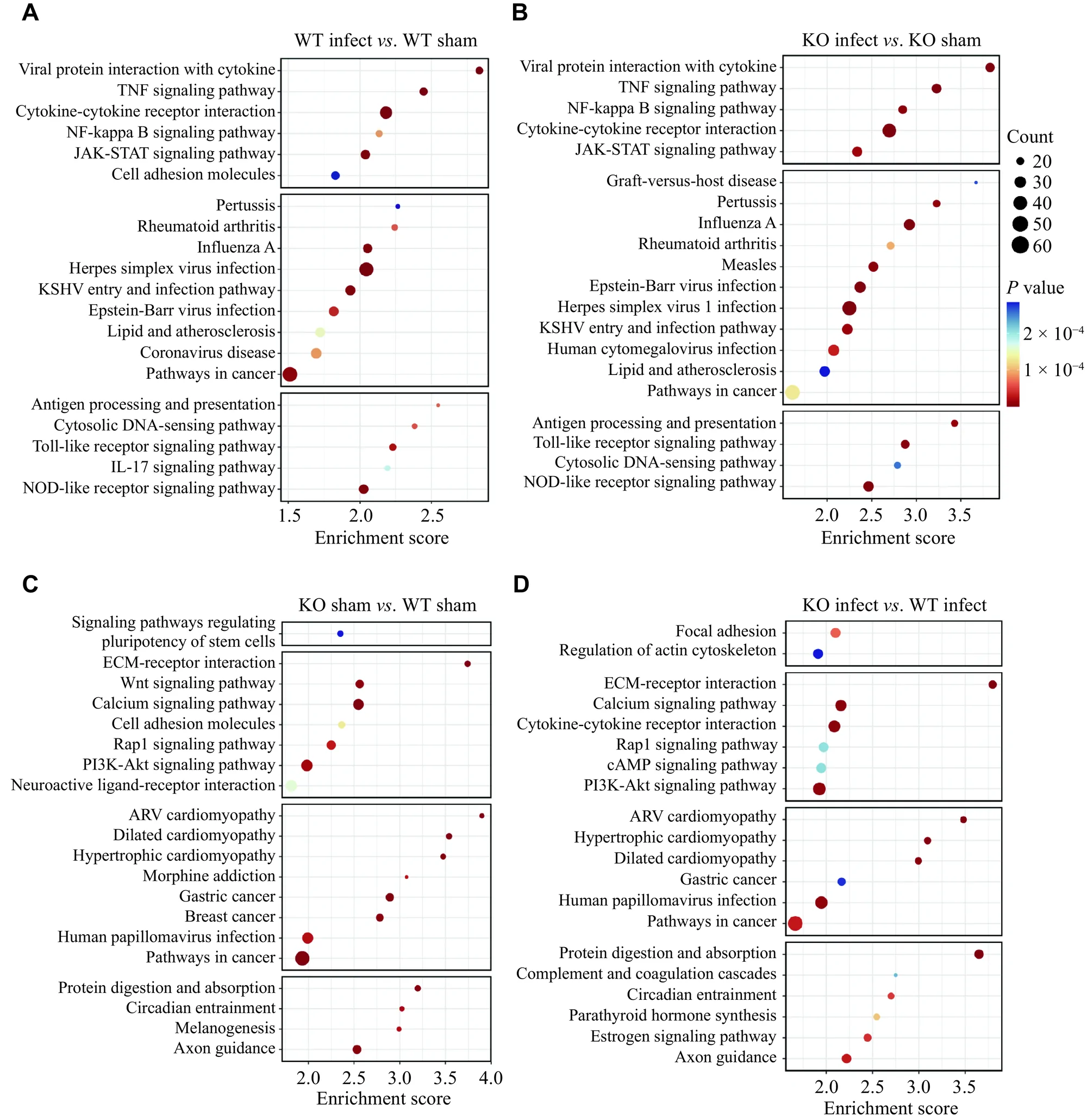
Transcriptome analyses of *ST6GAL1*^−/−^/*ST3GAL4*^−/−^ and wild-type (WT) porcine fibroblasts under H1N1 influenza A virus (IAV) infection. A and B: Kyoto Encyclopedia of Genes and Genomes (KEGG) pathway enrichment analysis of differentially expressed genes (DEGs) in WT (A) and knockout (KO) porcine fibroblasts (B) under sham and H1N1-infected treatments. C and D: KEGG pathway enrichment analysis of DEGs comparing KO and WT porcine fibroblasts under sham (C) and H1N1-infected (D) treatments. DEGs were filtered with |log_2_(fold change [FC]|) > 1 and *Q* < 0.05.

### Restoration of influenza virus susceptibility through gene complementation in *ST6GAL1*^−/−^/*ST3GAL4*^−/−^ Landrace pig fibroblasts

In the complementation experiments, we used the pCAGGS expression vector, which supports exogenous gene expression (***Supplementary Fig. 5***). Through homologous recombination technology, we constructed two complementation vectors, pCAGGS-*ST6* and pCAGGS-*ST3*, specifically designed for the *ST6GAL1* and *ST3GAL4* genes, respectively (***Supplementary Fig. 6***). These vectors were electroporated into *ST6GAL1*^−/−^/*ST3GAL4*^−/−^ Landrace pig fibroblasts to generate genetically complemented cells. Quantitative PCR analysis confirmed a significant upregulation of *ST6GAL1* and *ST3GAL4* mRNA expression in the complemented cells, achieving expression levels comparable to those in the WT controls (***Fig. 5A***). IF staining demonstrated the restoration of α2,6-linked and α2,3-linked sialylated glycan expression in the complemented cells (***Fig. 5B***). Upon challenge with the H1N1 virus, the complemented cells exhibited pronounced cytopathic effects, showing higher cell mortality compared with other groups (***Fig. 5C***). At 48 HPI, indirect immunofluorescence analysis revealed significantly stronger IAV detection signals in both complemented and WT cells compared with KO pig cells (***Fig. 5D***). These observations were quantitatively confirmed through two independent approaches: quantitative PCR demonstrated higher viral loads in the complemented cells compared with the KO group (***Fig. 5E***), and plaque assays showed a similar trend (***Fig. 5F***). Collectively, these results indicate that viral infection levels in complemented cells not only surpassed those in KO cells but also slightly exceeded those in wild-type controls, suggesting potential overexpression effects from the complementation system.

**Figure 5:**
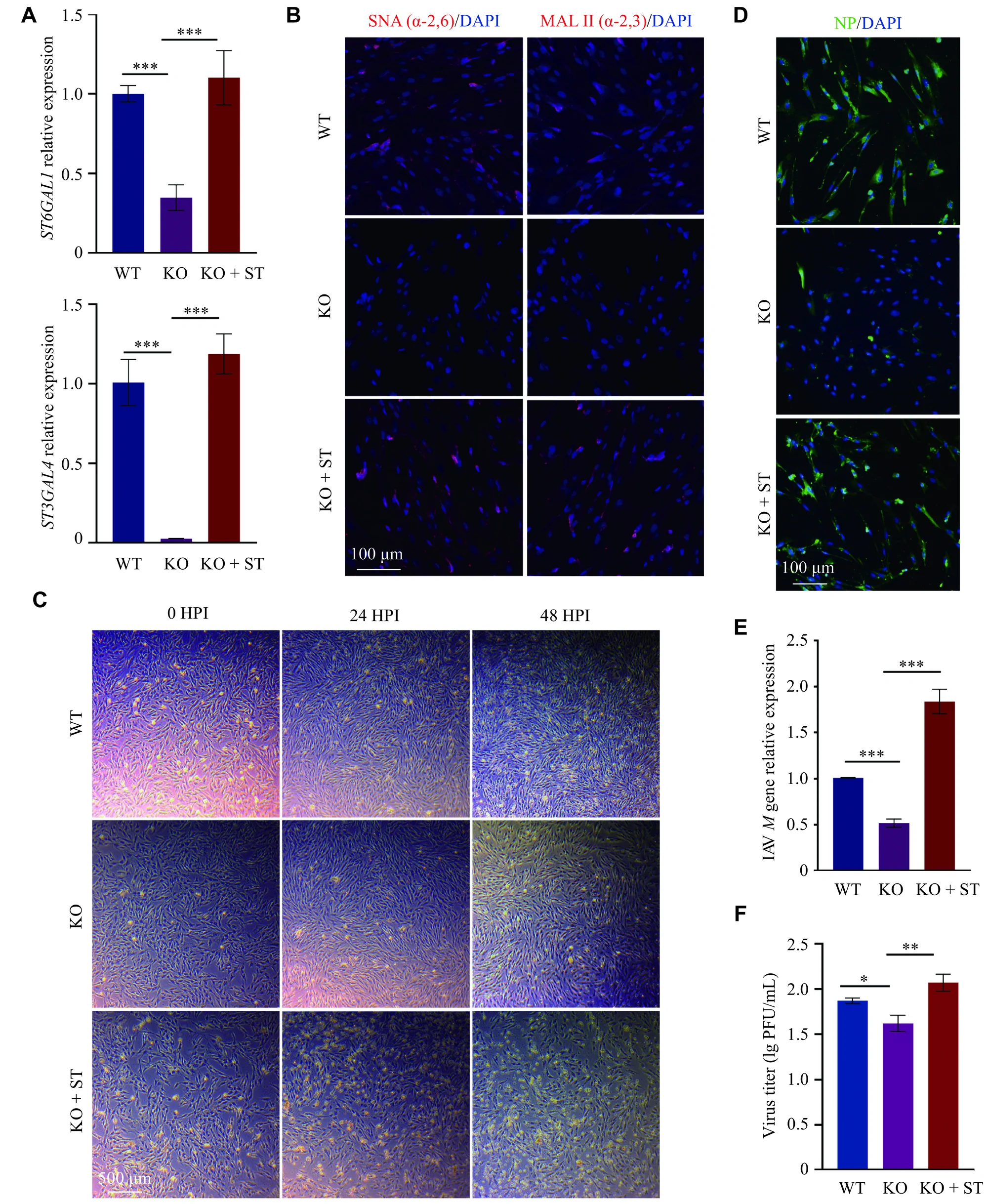
Restoration of influenza virus susceptibility in *ST6GAL1*^−/−^/*ST3GAL4*^−/−^ Landrace pig cells through complementation. A: Quantitative reverse transcription-PCR (RT-qPCR) analysis of *ST6GAL1* and *ST3GAL4* gene expression levels in wild-type (WT), knockout (KO), and complemented (KO + ST) cells. B: Immunofluorescent detection of α2,6-linked and α2,3-linked sialylated glycans in wild-type (WT), knockout (KO), and complemented (KO + ST) Landrace pig cells. Red indicates SNA (α2,6) or MAL Ⅱ (α2,3) staining, and blue indicates nuclei. The KO group showed nearly undetectable signals for both SNA and MAL Ⅱ. In contrast, the WT and KO + ST groups exhibited strong fluorescence for both lectins, indicating successful restoration of both α2,6- and α2,3-linked sialylated glycans in complemented cells. Scale bars, 100 μm. C: Cytopathic effects induced by H1N1 influenza virus infection at 0, 24, and 48 h post-infection (HPI). Scale bars, 500 μm. D: Immunofluorescent detection of IAV nucleoprotein (NP) in cells post-infection with H1N1 influenza virus at 48 HPI. NP-Alexa Fluor 488, green; DAPI, blue. Scale bars, 100 μm. E: RT-qPCR analysis of influenza viral *M* gene expression levels. F: Plaque assay measurement of infectious IAV (H1N1) virus titers (lg PFU/mL). Data are presented as mean ± standard deviation (A, E, and F); *n* = 3 independent cell culture experiments conducted on separate days with identical cell batches. Statistical significance was determined by one-way ANOVA followed by Tukey's multiple comparisons test. ^*^P < 0.05, ^**^P < 0.01, and ^***^P < 0.001.

## Discussion

Influenza A virus infection is influenced by multiple host and viral determinants, including HA-binding specificity, the distribution and structure of host glycans, proteolytic activation of viral proteins, intracellular replication factors, and innate immune responses. Pigs play an important role in interspecies transmission because they co-express both avian-type (α2,3-linked) and human-type (α2,6-linked) sialylated glycans in their respiratory tracts. Although dual sialylated glycan expression has been observed in several species—including white midget turkeys, bobwhite quails, pearl guinea fowl, chickens, and pheasants^[35–36]^, and even α2,3- and α2,6-linked sialylated glycans have been identified in human lungs^[37]^—the stable expression of both α2,3- and α2,6-linked sialylated glycans throughout the pigs' respiratory tract is particularly noteworthy. This consistent expression, combined with pigs' ecological role as frequent hosts for IAVs of diverse origins, makes them especially susceptible to coinfection and viral reassortment. When avian, human, or other mammalian influenza viruses co-infect the same pig cell, reassortment may occur, potentially leading to the emergence of novel viruses with new HA/NA antigenic combinations, to which the human population has little or no pre-existing immunity. It is the emergence of these antigenically distinct strains, capable of sustained human transmission, that poses a substantial pandemic threat, rather than the intrinsic virulence of the virus alone.

In our study, we knocked out the *ST6GAL1* and *ST3GAL4* genes in Landrace pigs to disrupt the expression of key sialylated glycans implicated in influenza virus binding. Despite the detection of residual mRNA due to frameshift mutations, immunofluorescence analysis confirmed the marked depletion of SNA- and MAL Ⅱ-reactive α2,3/α2,6 sialylated glycans across the porcine respiratory tract. Other studies have confirmed that in the porcine upper respiratory tract, α2,6-linked sialylated glycans are predominantly localized in pseudostratified ciliated columnar epithelial cells, exhibiting a continuous distribution from the trachea down to the bronchioles. In contrast, α2,3-linked sialylated glycan expression progressively increases along the lower respiratory epithelium and predominates in the submucosal layer of bronchi^[33–34]^. In our study, tissue IF staining revealed that the distribution of both sialylated glycans was nearly abolished in the larynx, trachea, and bronchi of KO pigs, with only a small amount scattered in pulmonary tissue, predominantly localized to the bronchiolar epithelium and endothelial cells. An interesting finding was the unexpected persistence of low-level α2,3-linked sialylated glycans in the bronchiolar epithelium of KO pig lung tissue. This residual expression may reflect compensatory upregulation of other ST3GAL family members following *ST3GAL4* knockout. In support of this hypothesis, RNA-seq analysis revealed a significant increase in *ST3GAL1* and *ST3GAL5* transcripts in KO pig cells, suggesting functional redundancy within the ST3GAL family (***Supplementary Fig. 4C***). This finding, together with the previously reported role of ST3GAL3 and ST3GAL6 in synthesizing Neu5Ac(α2-3)GAL linkages^[38]^, provides evidence for a coordinated compensatory network that maintains low levels of α2,3-linked sialylated glycans despite the absence of *ST3GAL4*. This potential compensatory mechanism requires further investigation to elucidate its molecular basis and biological significance. Importantly, the overall sialylated glycan knockout efficiency achieved in this study demonstrates the successful disruption of the major influenza virus binding sites throughout the porcine respiratory system.

Sialic-acid-containing glycans serve as HA attachment determinants for influenza A virus and also regulate various biological processes by glycosylating cell surface proteins and lipids, significantly contributing to both physiological and pathological immune responses. In the current study, no overt abnormalities were observed among the surviving KO pigs during the observation period (***Fig. 1C*** and ***Supplementary Fig. 7***). However, the small number of animals precludes definitive conclusions regarding long-term physiological stability, reproductive performance, or production-trait safety. Although postmortem analysis of the two deceased neonates revealed no anatomical abnormalities (***Fig. 1D***), the small sample size and absence of detailed molecular or histopathological analyses preclude determination of whether the deaths were related to SCNT, genetic modification, or other neonatal factors. Despite these unresolved safety concerns, our gene-targeting strategy was deliberately designed to limit off-target physiological effects. The choice to knock out the *ST3GAL4* and *ST6GAL1* genes was based on their distinctly high expression patterns in respiratory tissues compared with other sialyltransferase family genes^[39–40]^. Previous studies have shown that knocking out these two genes in airway epithelial cells significantly decreases IAV susceptibility while maintaining normal cellular viability^[31–32,41]^. This evidence strongly supported our rationale for targeting *ST3GAL4* and *ST6GAL1* to effectively reduce the expression of α2,6-linked sialylated glycans and α2,3-linked sialylated glycans in the porcine respiratory system, while minimizing potential impacts on other physiological functions.

Our viral challenge assays in fibroblasts derived from *ST6GAL1*^−/−^/*ST3GAL4*^−/−^ pigs demonstrated a substantial reduction in viral infection, replication, and cytopathic effects compared with wild-type controls. The gene complementation rescue experiment strongly supports a causal contribution of ST6GAL1/ST3GAL4-dependent sialylation to IAV susceptibility. We also note that complementation vectors drive exogenous gene overexpression, resulting in moderately higher viral loads relative to wild-type cells and precluding complete physiological restoration.

Nevertheless, the absence of complete protection suggests the involvement of alternative entry pathways. We propose that residual infectivity may be attributed to functional compensation by other sialyltransferases and the utilization of non-canonical receptors. Byrd-Leotis *et al*^[37,42]^ have identified phosphorylated glycans in porcine and human lungs as potential attachment factors for IAVs by glycomics. In our KO pigs, these phosphorylated glycans may act as alternative attachment factors, potentially compensating for the loss of α2,3- and α2,6-linked sialylated glycans and accounting for the residual viral infectivity observed. Furthermore, the recent identification of metabotropic glutamate receptor subtype 2 (mGluR2) as a bona fide viral entry receptor for IAVs adds another layer of complexity^[43]^. Unlike glycan-based attachment factors that only mediate superficial HA binding, mGluR2 mediates clathrin-dependent endocytosis to complete viral internalization, and this process operates independently of sialylated glycan ligands. Its glycosylation is dispensable for interaction with HA, suggesting that it may function as a fail-safe mechanism for viral entry under conditions with limited canonical glycan attachment sites. It is well documented that selective pressure can remodel the HA glycan-binding pocket. Serial viral propagation in glycan-attachment-limited environments, such as our KO pigs, could select for variants with enhanced affinity for phosphorylated glycans or protein-based receptors like mGluR2. Such adaptations could mitigate the protective effect of ablation of α2,3/α2,6 sialylated glycans and underscore the need for multi-target intervention strategies. Future studies should monitor viral evolution in this model to evaluate the potential emergence of receptor-switching variants.

Transcriptomic profiling revealed a marked reduction in DEGs between knockout and WT cells post-infection compared with pre-infection states. This attenuated differential response may be attributed to either a reduced viral infection load in the knockout group or the suppression of virus-infection-related signaling pathways caused by the knockout of *ST6GAL1* and *ST3GAL4* genes, both of which reflect the protective effect of dual sialyltransferase ablation against IAV infection at the transcriptional level. KEGG pathway analysis showed that the DEGs were mainly enriched in pathways associated with cancer and cardiomyopathies. This enrichment was consistent with the known involvement of sialylation-related processes in diverse cellular functions; however, pathway enrichment alone does not validate successful gene knockout and should be interpreted as hypothesis-generating. Genotyping, transcript analysis, lectin staining, and the cellular infection phenotype provide the primary evidence for validation of the edited model^[44–46]^. Additionally, persistent enrichment of DEGs in signal transduction pathways in knockout cells, both pre- and post-infection, suggests these sialyltransferases regulate cellular information processing and environmental response mechanisms. Specifically, H1N1-infected KO pig cells exhibited distinct enrichment in the complement and coagulation cascade pathway compared with the WT group, which may affect the efficiency of virus clearance or the intensity of the inflammatory response. These changes reflect the differences in host defense mechanisms triggered by sialylated glycan deficiency. The findings identify *ST6GAL1/ST3GAL4* as potential targets for further exploring the mechanistic interplay between viral infection and host glycosylation modification in immune response regulation.

Several limitations of the current study should be acknowledged. First, IAV susceptibility was assessed in cultured porcine fibroblasts rather than by direct viral challenge of live gene-edited pigs. Therefore, the effects of *ST6GAL1/ST3GAL4* deletion on infection, clinical disease, viral shedding, intraswine transmission, or cross-species transmission remain unknown. Second, only five cloned male piglets were generated, two of which died during the neonatal period, leaving three surviving animals for long-term observation. This limited cohort precludes definitive conclusions regarding long-term physiological, reproductive, immunological, metabolic, or production-trait consequences. Third, all five piglets originated from the same donor-cell clone, and phenotypic replication across independently generated edited founder lines was therefore not assessed. Fourth, comprehensive genome-wide off-target sequencing and systematic validation of predicted high-risk off-target sites were not performed in this proof-of-concept study. Consequently, unintended genomic alterations cannot be excluded. Fifth, the viral challenge experiments were conducted in fibroblasts rather than primary respiratory epithelial cells, which may not fully reproduce the biology of the porcine airway. Finally, the possible compensatory effects of other sialyltransferases and alternative influenza virus attachment or entry factors require further investigation.

Future studies should include direct *in vivo* viral challenge of independently generated edited pigs using representative IAV strains, together with longitudinal assessments of clinical signs, viral load, tissue distribution, viral shedding, and transmission between animals. Primary porcine airway epithelial cells, airway organoids, and *ex vivo* respiratory tissues may help bridge the gap between fibroblast assays and whole-animal infection.

Comprehensive genomic analysis, including assessment of predicted high-risk off-target loci and, where feasible, whole-genome sequencing, will also be required. Detailed glycomic profiling should be performed to characterize residual α2,3- and α2,6-linked structures and determine whether alternative attachment factors or entry pathways contribute to residual infection. Long-term reproductive, immunological, metabolic, developmental, and production-trait assessments in larger, independently generated cohorts will be necessary to evaluate the physiological consequences of the edited genotype.

In summary, we generated *ST6GAL1/ST3GAL4* double-knockout Landrace pigs with markedly reduced detectable α2,6- and α2,3-linked sialylated glycans in the respiratory tract. Fibroblasts derived from these pigs showed reduced susceptibility to H1N1, H3N2, and H9N2 influenza A virus infection *in vitro*, and complementation of *ST6GAL1* and *ST3GAL4* restored sialylated glycan expression and increased viral susceptibility. These findings support the use of this model for investigating host determinants of influenza virus attachment and susceptibility. However, direct *in vivo* viral challenge, transmission studies, validation in independently generated edited lines, comprehensive off-target assessment, and long-term physiological evaluation are required before the potential of this strategy to reduce influenza transmission can be determined.

## Additional information

The online version contains supplementary material available at http://www.jbr-pub.org.cn/article/doi/10.7555/JBR.39.20250300.
