# Supplementary material for: Generation of ST6GAL1/ST3GAL4 double-knockout Landrace pigs with reduced respiratory sialylated glycans and decreased influenza A virus susceptibility of derived fibroblasts in vitro
Source: J Biomed Res. 2026 Sep 28;40(5):505–20. doi: 10.7555/JBR.39.20250300 (PMC13620455; doi:10.7555/JBR.39.20250300)
Supplement: Supplementary file 1 [file jbr-40-5-505-S1.pdf]

Available online at www.jbr-pub.org.cn

Open Access at PubMed Central

Journal of Biomedical Research, 2026 40(5): 1–6

Supplementary Data

JBR

Generation of *ST6GAL1/ST3GAL4* double-knockout Landrace pigs with reduced respiratory sialylated glycans and decreased influenza A virus susceptibility of derived fibroblasts *in vitro*

Lin Li[1,2], Shuyi Fang[1,2], Xiaoxue Li[1,2], Jingjing Liu[1,2], Wenhan Lu[1,2], Qinyuan Li[1,2], Jianghao Chang[1,2], Yilin Yuan[3], Bin Fang[3], Ying Wang[1,2], Yi Rao[4,✉], Haiyuan Yang[1,2,✉], Yifan Dai[1,2,✉]

[1]*Department of Medical Genetics, School of Basic Medical Science, Nanjing Medical University, Nanjing, Jiangsu 211166, China;*
[2]*Jiangsu Province Key Laboratory of Biological Therapy for Organ Failure, Nanjing Medical University, Nanjing, Jiangsu 211166, China;*
[3]*Gcreatene (Suzhou) Biotechnology Co., Ltd., Suzhou Industrial Park, Suzhou, Jiangsu 215000, China;*
[4]*Peking-Tsinghua Center for Life Sciences, Peking University, Beijing 100871, China.*

Supplementary Table 1 **Primer sequences used for *ST6GAL1* and *ST3GAL4* gene amplification**

Gene name	Sequence (5′–3′)
ST6GAL1	Forward: AGGGTCCCCATGCTCAAAAT
	Reverse: TTAGCTGGCACTTGCCTCAC
ST3GAL4	Forward: CAAGAACAGCAACGAGTACCG
	Reverse: ACAGCCCTATGCTTGGGGTA
pCAGGS-*ST6*	Forward: TTTGGCAAAGAATTCGGGGGAATGATTCACACCAGCTTGA
	Reverse: AGGGAAAAAGATCTGGTTATTTCATGCTCCACAGTGAA
pCAGGS-*ST3*	Forward: TTTGGCAAAGAATTCGGGGGAATGATCAGCAAGTGCCGCT
	Reverse: AGGGAAAAAGATCTGGTTATTTCAGAAGTATGTGAGGTT

Supplementary Table 2 **Primer sequences used for quantitative polymerase chain reaction**

Gene name	Forward (5′–3′)	Reverse (5′–3′)
ST6GAL1	CCACCCGCTACTCTTTGAGA	AAATACCCAGTGGAGAAGGGC
ST3GAL4	ACTCCTCGTCCTGGTAGCTT	ATCGGCAGCCTTAGCAGTTT
GAPDH	GCAAAGTGGACATTGTCGCCATCA	TCCTGGAAGATGGTGATGGCCTTT

✉Corresponding authors: Yi Rao, E-mail: yrao@pku.edu.cn, ORCID: 0000-0002-0405-5426; Haiyuan Yang, E-mail: hyyang@njmu.edu.cn, ORCID: 0000-0001-5253-0079; Yifan Dai, E-mail: daiyifan@njmu.edu.cn, ORCID: 0000-0003-2872-8442.

Received: 14 July 2025; Revised: 14 November 2025; Accepted: 19 November 2025; Available online: 25 November 2025; Published date: 28 September 2026

CLC number: R373.1, Document code: A

The authors reported no conflicts of interest.

This is an open access article under the Creative Commons Attribution (CC BY 4.0) license, which permits others to distribute, remix, adapt and build upon this work, for commercial use, provided the original work is properly cited.

© 2026 by Journal of Biomedical Research.

https://doi.org/10.7555/JBR.39.20250300

Supplementary Table 3 **Genotypes of *ST6GAL1* biallelic mutation clones**

No.	Sequence	Genotype/bp
Wild-type	ATCCCGACTACCGTTTCTTCGATAACTT……CATCATTCAAGAATCTCCCCAGAGGCCATCCAGCC	WT
5	ATCCCGACTACCGTTTCTTCGATAACTT……CATCATTCAAGAAATCTCCCACAGAGGCCATCCAGCC	+1
5	ATCC--AGCC	△131
7	ATCCCGAC---CAGAGGCCATCCAGCC	△115
8	ATCCCGAC---CAGAGGCCATCCAGCC	△115
8	ATCCCGACT---CCCCAGAGGCCATCCAGCC	△111
23	ATCCCGACTACCGTTTCTTCGATAACTT……CATCATTCAAG AAATCTCC----AGAGGCCATCCAGCC	△2
24	ATCCCGACTTACCGTTTCTTCGATAACTT……CATCATTCAAGAAATCTCCC-AGAGGCCATCCAGCC	+1△1
32	ATCCCGAC---CAGAGGCCATCCAGCC	△115
33	ATCCCGAC---CAGAGGCCATCCAGCC	△115
36	ATCCCGAC---CAGAGGCCATCCAGCC	△115
37	ATCCCGAC---CAGAGGCCATCCAGCC	△115
38	ATCCCGAC---CCAGAGGCCATCCAGCC	△114
41	ATCCCGAC---CCAGAGGCCATCCAGCC	△114
46	ATCCCGAC---CAGAGGCCATCCAGCC	△115
62	ATCCCGAC---CAGAGGCCATCCAGCC	△115
63	ATCCCGACTACCGTTTCTTCGATAACTT……--------------------------------------GCCATCCAGCC	△33
66	ATCCCGACTTACCGTTTCTTCGATAACTT……CATCATTCAAGAATCTCCC--AGAGGCCATCCAGCC	+1△1
68	ATCCCGAC--CCCAGAGGCCATCCAGCC	△112
78	ATCCCGACTTACCGTTTCTTCGATAACTT……CATCATTCAAGAAATCTCCCCCAGAGGCCATCCAGCC	+2

Supplementary Table 4 **Genotypes of *ST3GAL4* biallelic mutation clones**

No.	Sequence	Genotype/bp
Wild-type	AGGCTCAAGTGCCGCCGCTGCGTGGTGG……GGACTTCCACTGGATTGAGAGCATCTTGAGTG	WT
5	AGGCTCAAGTGCCGCCGCTGCGTGGTGG……GGACTTCCACTGGA---------------ATCTTGAGTG	△8
5	AGGCTCAAGTGCCGCCGCTTGCGTGGTGG……GGACTTCCACTGGATTGAGAGCATCTTGAGTG	+1
7	AGGCTCAAGTGCCGCCGCTGCGTGGTGG……GGACTTCCACTGAGATTGAGAGCATCTTGAGTG	+1
8	AGGCTCAAGTGCCGCCGCTGCGTGGTGG……GGACTTCCACTG--ATTGAGAGCATCTTGAGTG	△1
23	AGGCTCAAGTGCCGCCGCTGCGTGGTGG……GGACTT---GTG	△26
24	AGGCTCAAGTGCCGCCGCTGCGTGGTGG……GGACTTCCACTG--ATTGAGAGCATCTTGAGTG	△1
24	AGGCTCAAGTG---ATTGAGAGCATCTTGAGTG	△333
32	AGGCTCAAGTGCCGCCGCTGCGTGGTGG……GGACTTCCACTG--ATTGAGAGCATCTTGAGTG	△1
33	AGGCTCAAGTGCCGCCGCTGCGTGGTGG……GGACTTCCACTG--ATTGAGAGCATCTTGAGTG	△1
36	AGGCTCAAGTGCCGCCGCTGCGTGGTGG……GGACTTCCACTGGGATTGAGAGCATCTTGAGTG	+1
37	AGGCTCAAGTGCCGCCGCTGCGTGGTGG…---TCTTGAGTG	△52
38	AGGCTCAAGTGCCGCCGCTGCGTGGTGG……GGACTTCCACTG-----TTGAGAGCATCTTGAGTG	△2
41	AGGCTCAAGTGCCGCCGCTGCGTGGTGG……GGACTTCCACTG----ATTGAGAGCATCTTGAGTG	△1
46	AGGCTCAAGTGCCGCCGCTGCGTGGTGG……GGACTTCCACTGTGATTGAGAGCATCTTGAGTG	+1
62	AGGCTCAAGTGCCGCCGCTGCGTGGTGG……--	△45
63	AGGCTCAAGTGCCGCCGCTGCGTGGTGG……G-----------------------------AGAGCATCTTGAGTG	△16
66	AGGCTCAAGTG---ATTGAGAGCATCTTGAGTG	△333
68	AGGCTCAAGTGCCGCCGCTTGCGTGGTGG……GGACTTCCACTG--ATTGAGAGCATCTTGAGTG	+1△1
78	AGGCTCAAGTGCCGCCGCTGCGTGGTGG……GGACTTCCACTG---------AGAGCATCTTGAGTG	△5

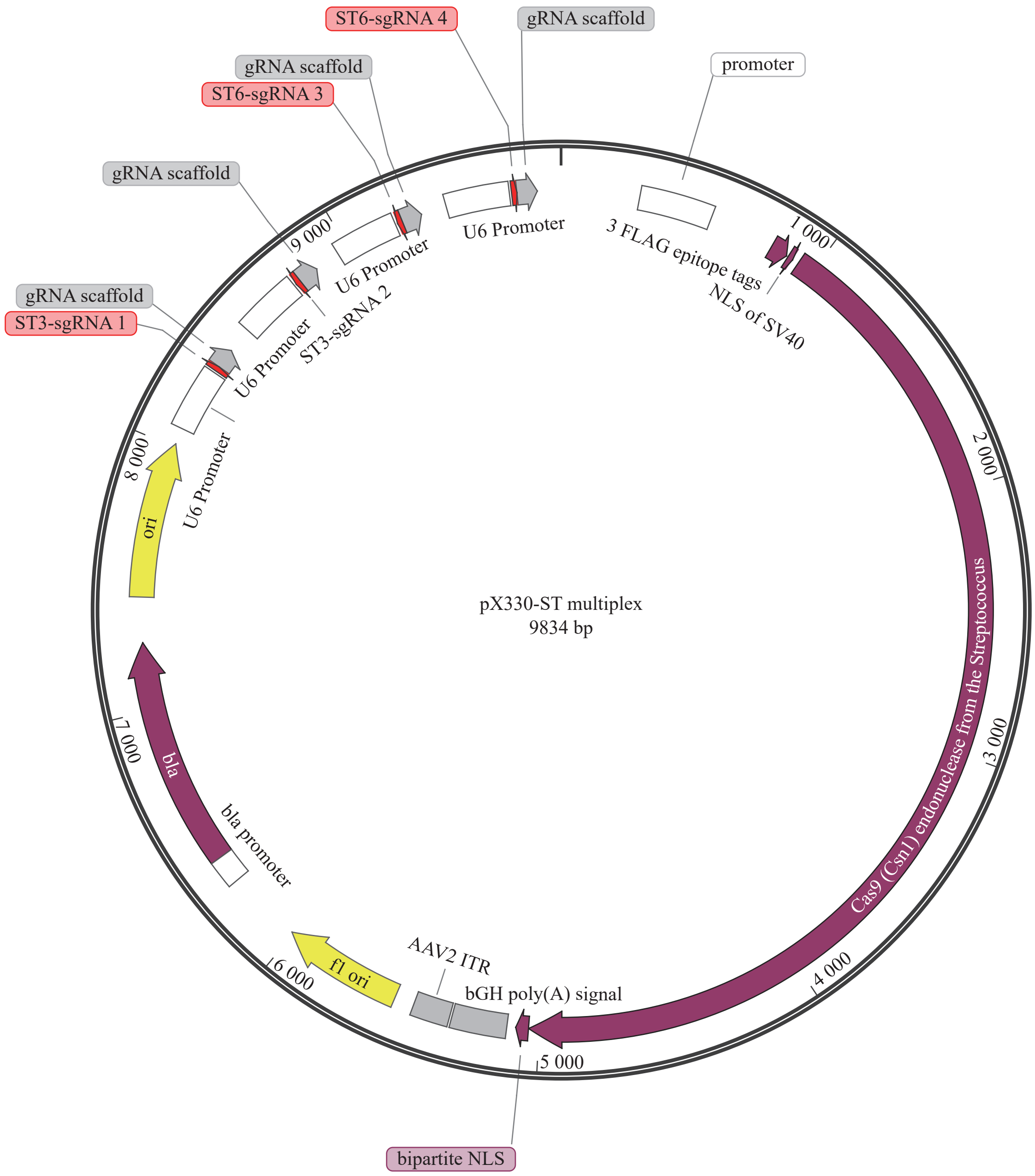

Supplementary Fig. 1 **Plasmid map of pX330-ST vector.**

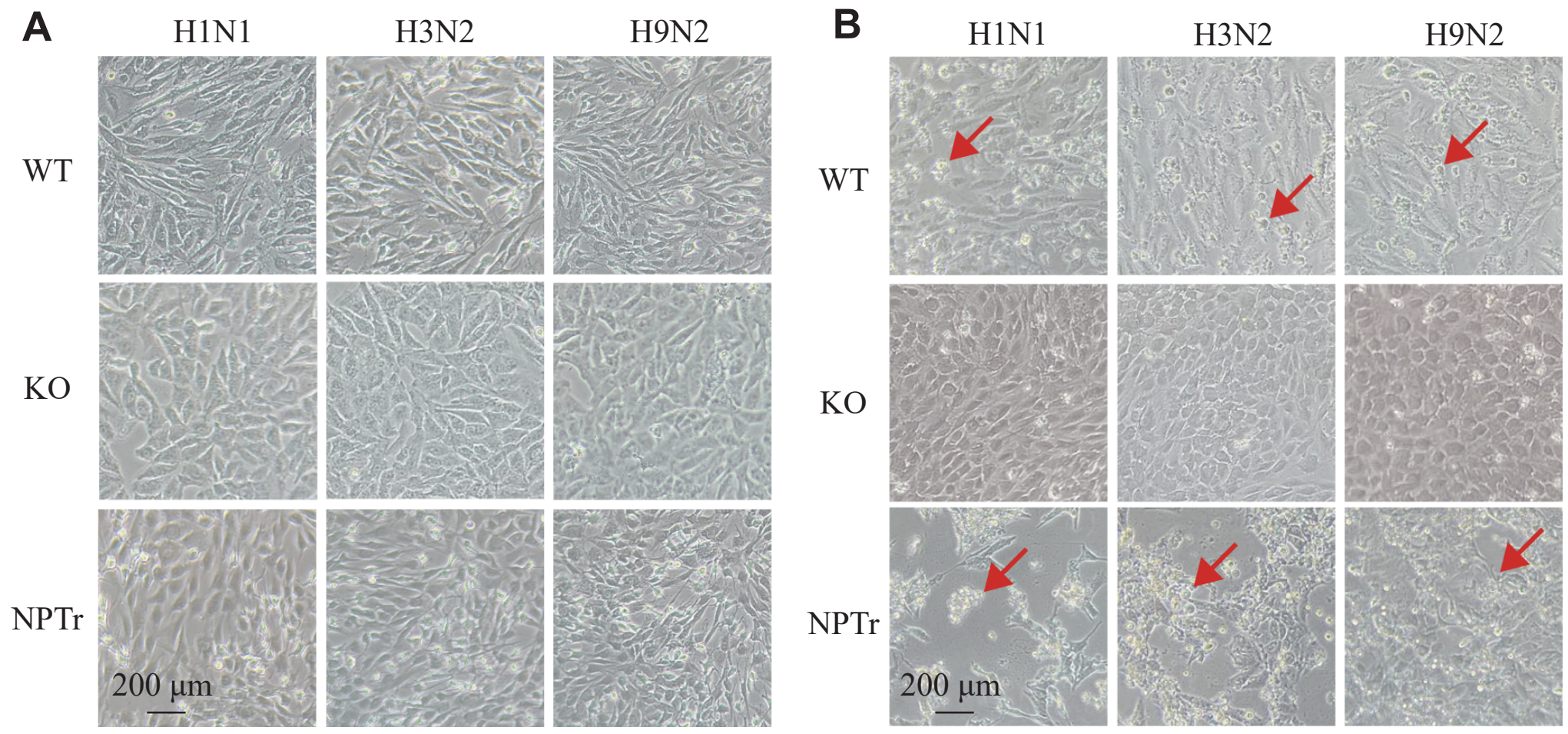

Supplementary Fig. 2 **Cytopathic effects induced by H1N1, H3N2, and H9N2 influenza A virus infection.** A: Uninfected wild-type pig fibroblasts (WT), knockout pig fibroblasts (KO) and newborn pig trachea epithelial cells (NPTr). B: Cells at 72 h post-infection. Both the wild-type fibroblasts (WT) and newborn pig trachea epithelial (NPTr) cells groups exhibited significant cytopathic effects (red arrows), with the NPTr group showing extensive cell death and detachment, particularly pronounced in H1N1-infected samples. Scale bar, 200 μm.

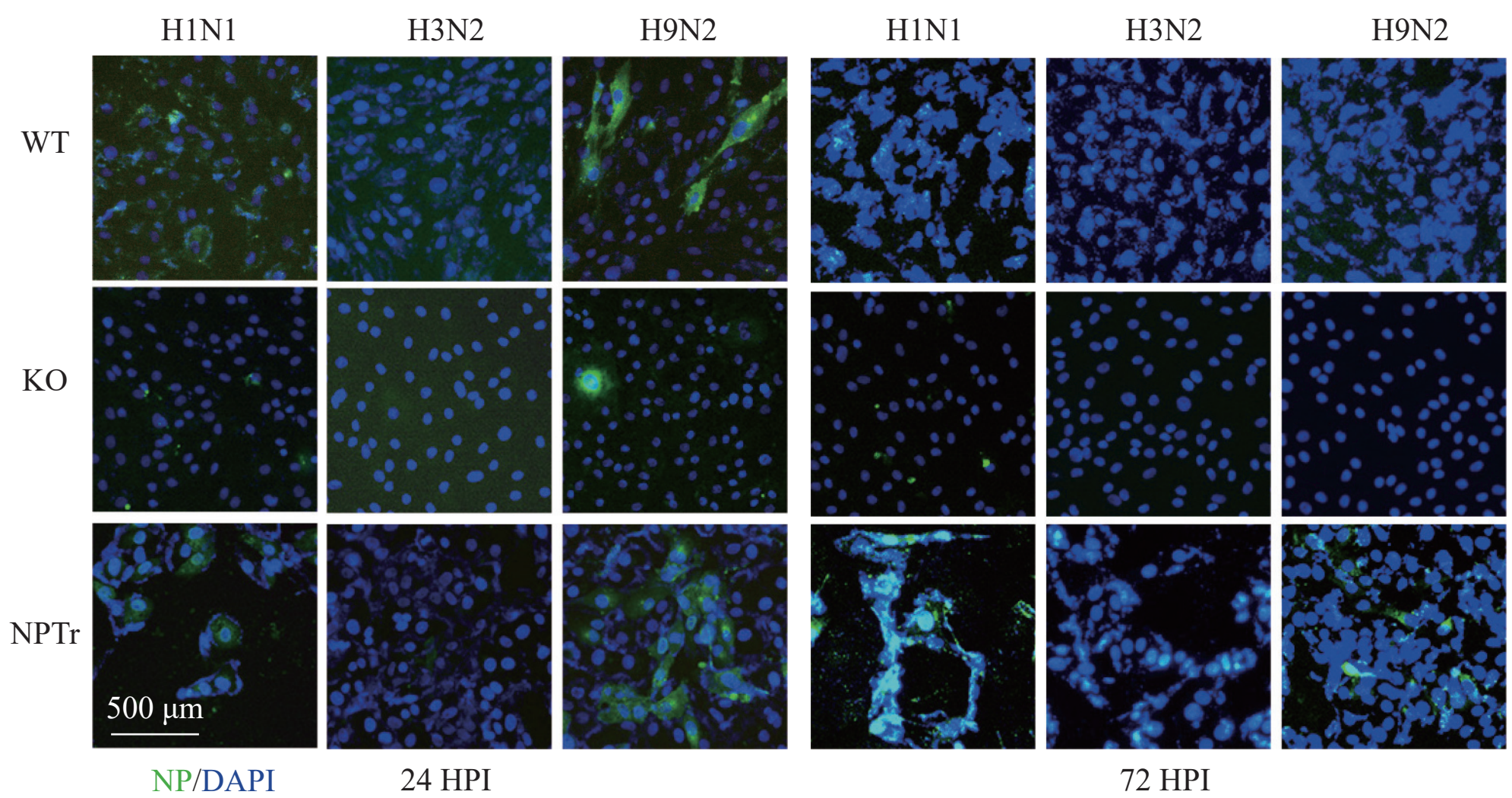

Supplementary Fig. 3 **Immunofluorescent detection of influenza A virus (IAV) nucleoprotein (NP) in knockout (KO) and wild-type (WT) pig fibroblasts.** Cells were infected with H1N1, H3N2, or H9N2 strains of IAV and fixed at 24 and 72 h post-infection (HPI). Scale bar, 500 μm. Newborn pig trachea epithelial (NPTr) were used as positive control. NP-Alexa Fluor 488, green; DAPI, blue.

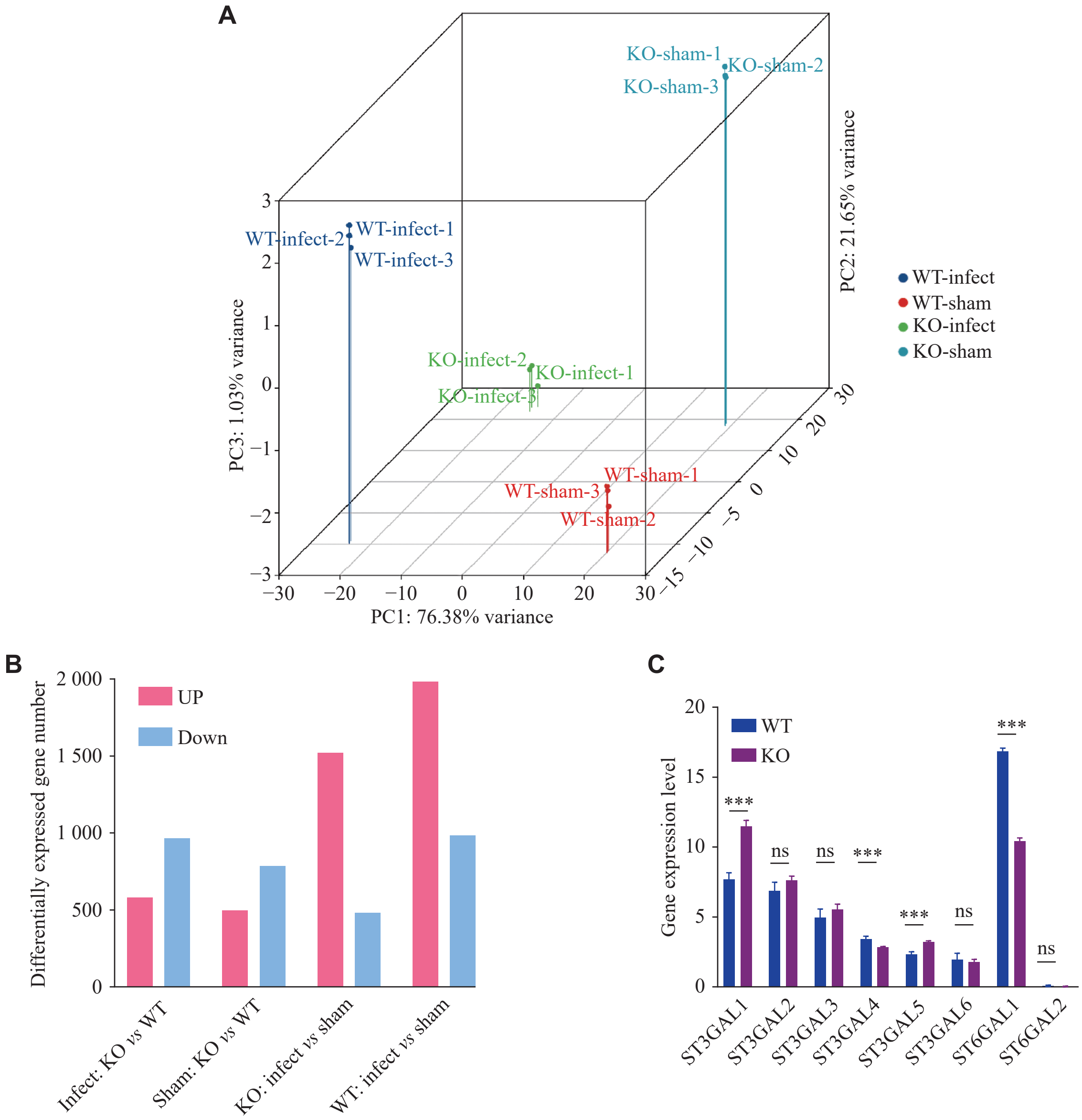

Supplementary Fig. 4 **Transcriptome analyses of *ST6GAL1*$^{-/-}$/*ST3GAL4*$^{-/-}$ and wild-type (WT) cells under H1N1 influenza A virus (IAV) infection.** A: Principal component analysis (PCA) of the transcriptome in knockout (KO) and WT porcine fetal fibroblasts (PFFs) upon infection (multiplicity of infection [MOI] = 0.5). B: Bar chart representing differentially expressed genes (DEGs). C: Gene expression profiles of *ST3GAL* and *ST6GAL* family members from RNA-seq analysis. Data are presented as mean ± standard deviation (n = 3 independent experiments denotes three fully separate cell-culture and infection procedures using the same cell strains) with statistical significance determined by one-way analysis of variance followed by Tukey's multiple-comparison test. ns, not significant, $^{**}P < 0.01$ and $^{***}P < 0.001$.

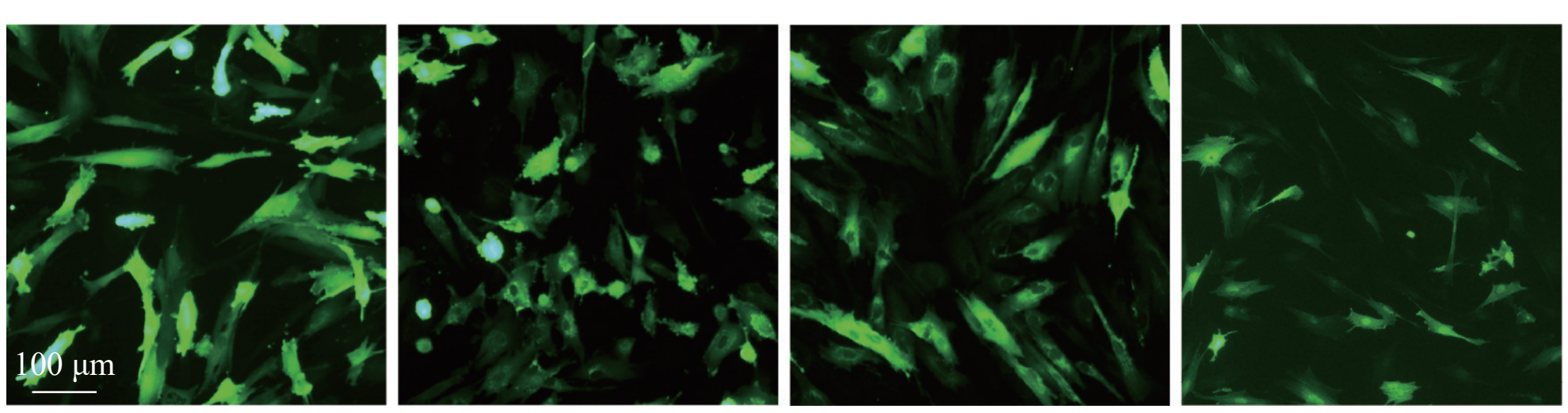

Supplementary Fig. 5 **Fluorescence detection of GFP protein in porcine fibroblast cells transfected with the pCAGGS-GFP plasmid.** The images (from left to right) show GFP expression at 1, 3, 6, and 10 days post-transfection. Scale bar, 100 μm.

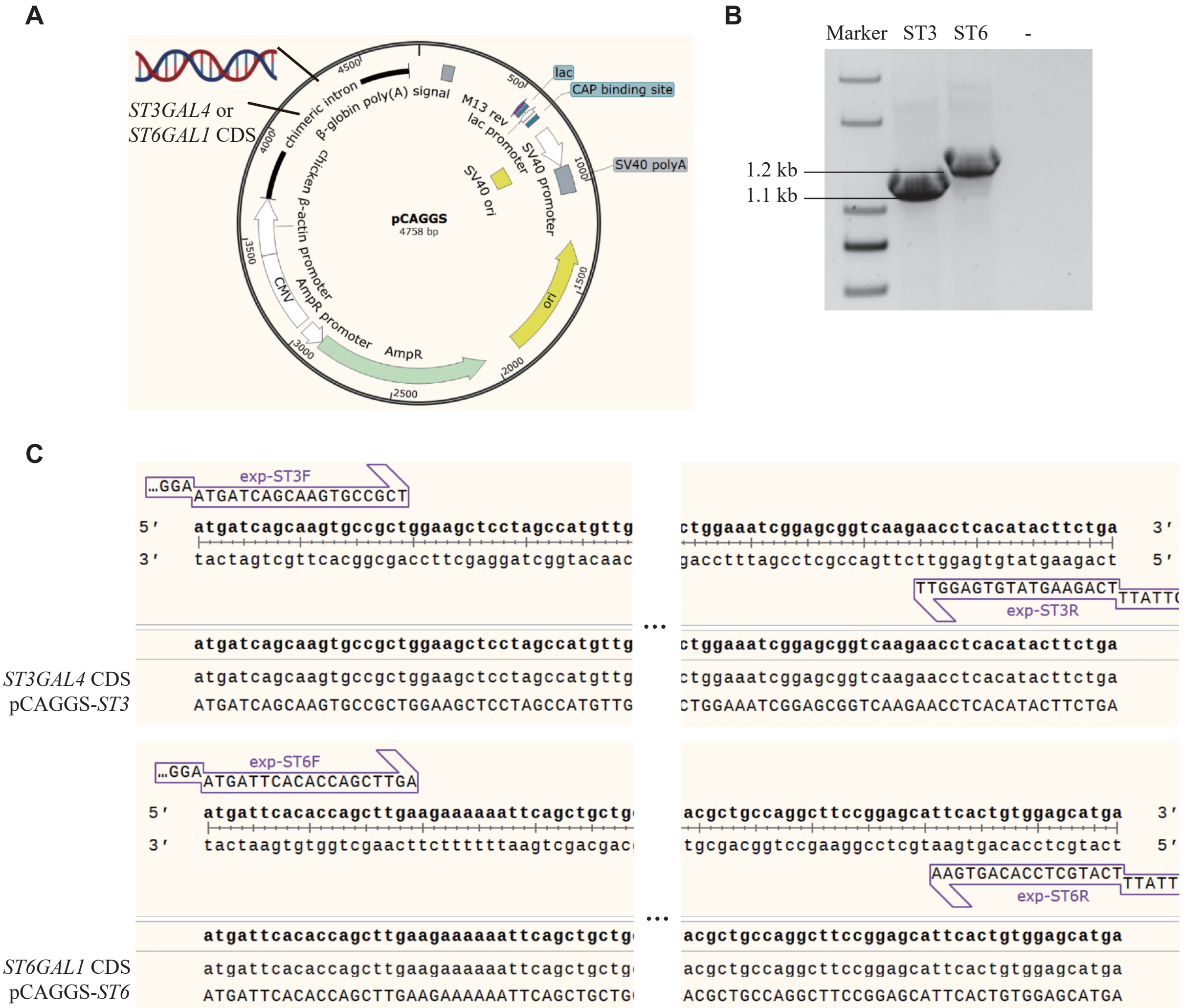

Supplementary Fig. 6 **Construction of the pCAGGS-*ST3* and pCAGGS-*ST6* expression vectors.** A: Schematic diagram of the pCAGGS-*ST3* and pCAGGS-*ST6* expression vectors. B: PCR results of the vectors. C: Partial sequencing results of the vectors, with the purple boxes highlighting the primer sequences containing homologous arms.

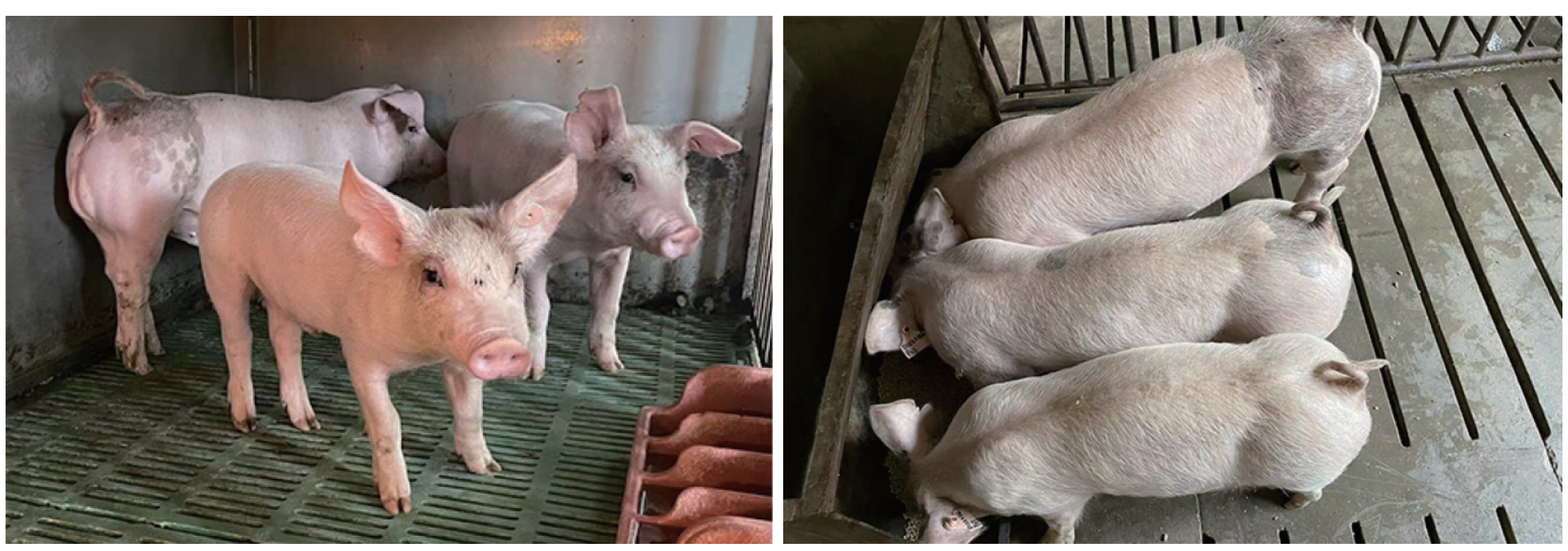

Supplementary Fig. 7 **Photographs of *ST6GAL1*$^{-/-}$/*ST3GAL4*$^{-/-}$ Landrace pigs at 3 (left) and 4 (right) months of age.**
